# ALKBH7 drives a tissue and sex-specific necrotic cell death response following alkylation-induced damage

**DOI:** 10.1038/cddis.2017.343

**Published:** 2017-07-20

**Authors:** Jennifer J Jordan, Sophea Chhim, Carrie M Margulies, Mariacarmela Allocca, Roderick T Bronson, Arne Klungland, Leona D Samson, Dragony Fu

**Affiliations:** 1 Department of Biological Engineering, Biology, Center for Environmental Health Sciences, Massachusetts Institute of Technology, Cambridge, MA, USA; 2 Department of Biology, University of Rochester, Rochester, NY, USA; 3 Rodent Histopathology Core, Harvard Medical School, Boston, MA, USA; 4 Department of Molecular Microbiology A3.3021, Oslo University Hospital, Rikshospitalet, Oslo, Norway

## Abstract

Regulated necrosis has emerged as a major cell death mechanism in response to different forms of physiological and pharmacological stress. The AlkB homolog 7 (ALKBH7) protein is required for regulated cellular necrosis in response to chemotherapeutic alkylating agents but its role within a whole organism is unknown. Here, we show that ALKBH7 modulates alkylation-induced cellular death through a tissue and sex-specific mechanism. At the whole-animal level, we find that ALKBH7 deficiency confers increased resistance to MMS-induced toxicity in male but not female mice. Moreover, ALKBH7-deficient mice exhibit protection against alkylation-mediated cytotoxicity in retinal photoreceptor and cerebellar granule cells, two cell types that undergo necrotic death through the initiation of the base excision repair pathway and hyperactivation of the PARP1/ARTD1 enzyme. Notably, the protection against alkylation-induced cerebellar degeneration is specific to ALKBH7-deficient male but not female mice. Our results uncover an *in vivo* role for ALKBH7 in mediating a sexually dimorphic tissue response to alkylation damage that could influence individual responses to chemotherapies based upon alkylating agents.

Certain environmental or pathological conditions can trigger a regulated form of necrotic cell death characterized by cytoplasmic swelling, vacuolization and rupture of the plasma membrane with subsequent stimulation of the inflammatory response (reviewed in references 1, 2, 3, 4, 5). Known triggers of regulated necrosis include pathogen infection and the innate immune response,^6, 7^ ischemia reperfusion^8^ or excessive DNA damage induced by alkylating or oxidizing agents.^9^ These triggers can initiate a diversity of overlapping as well as distinct necrotic cell death pathways including necroptosis, ferroptosis, parthanatos and pyroptosis.^5^ Although different necrotic cell death pathways have emerged, many of the protein factors that modulate regulated necrosis remain to be elucidated, including their cell and tissue specificities.

Chemotherapeutic alkylating agents can trigger a regulated form of necrosis that is dependent upon hyperactivation of poly-ADP-ribose polymerase 1 (PARP1; also known as ADP-ribosyltransferase and diphtheria toxin-like 1, ARTD1).^10, 11, 12, 13, 14^ PARP1/ARTD1-dependent cell death, also known as parthanatos, has also been shown to have a major role in cell death induced by physiological conditions such as ischemia reperfusion, Parkinson’s and uncontrolled diabetes (reviewed in Fatokun *et al.*^15^and Ying and Padanilam^16^). Alkylating agent-induced necrosis is characterized by the aforementioned PARP1/ARTD1 hyperactivation, subsequent intracellular NAD^+^ depletion, loss of ATP production, mitochondria depolarization, generation of reactive oxygen species (ROS) and eventual loss of membrane integrity.^17, 18, 19, 20, 21, 22^ In addition, alkylation damage can induce the translocation of apoptosis-inducing factor (AIF) from the mitochondria to the nucleus, where AIF forms a DNA-degrading complex with histone H2AX and cyclophilin D.^10, 23^ Alkylation-induced necrosis appears to be distinct from caspase-dependent apoptosis or receptor-mediated necroptosis as neither caspase nor necroptosis inhibitors can rescue alkylation-induced necrotic cell death.^24, 25, 26, 27^

We have previously shown that human AlkB homolog 7 (ALKBH7) is a mitochondrial protein required for PARP1/ARTD1-dependent regulated necrosis stimulated by alkylating and oxidizing agents.^28^ ALKBH7 is a mammalian ortholog of *Escherichia coli* AlkB, the founding member of the AlkB family of 2-oxoglutarate and iron-dependent dioxygenases (reviewed in Fedeles *et al.*^29^, Ougland *et al.*^30^ and Shen *et al.*^31^). ALKBH7 has a role downstream of PARP1/ARTD1 hyperactivation and NAD+ depletion in the regulated necrotic pathway by suppressing the recovery of cellular bioenergetics and triggering the final stages of necrosis. Thus, human cells depleted of ALKBH7 are resistant to regulated necrotic cell death induced by alkylating and oxidizing agents. In addition to necrosis, we have also shown that ALKBH7 has a role in fatty acid metabolism and obesity in mice.^32^ Mice deficient in ALKBH7 display increased body weight because of increased body fat as well as alterations in the levels of certain circulating triglycerides. Interestingly, a recent study has also identified a cancer-associated ALKBH7 mutation in humans that affects substrate binding in ALKBH7.^33^ Collectively, these studies indicate that ALKBH7 has multiple cellular roles by modulating necrotic cell death and metabolism.

Here, we investigated the biological role of ALKBH7 in mammalian cells and tissues after alkylating agent exposure using ALKBH7-knockout models. Using CRISPR-mediated genetic ablation, we show that loss of ALKBH7 expression results in protection against alkylation and oxidation-induced necrotic cell death. In addition to human and mouse cells, we demonstrate that ALKBH7 has a key role in mediating whole-body and tissue-specific toxicity in response to alkylation damage. Surprisingly, we find that ALKBH7-dependent effects on tissue necrosis is influenced by sex-specific mechanisms. Our results uncover a key link between a specific necrotic cell death factor and sex that could influence individual responses to therapeutic approaches based upon alkylating agents.

## Results

### ALKBH7-deficient human or mouse cells are resistant to cell death induced by alkylating agents

**Figure 1:**
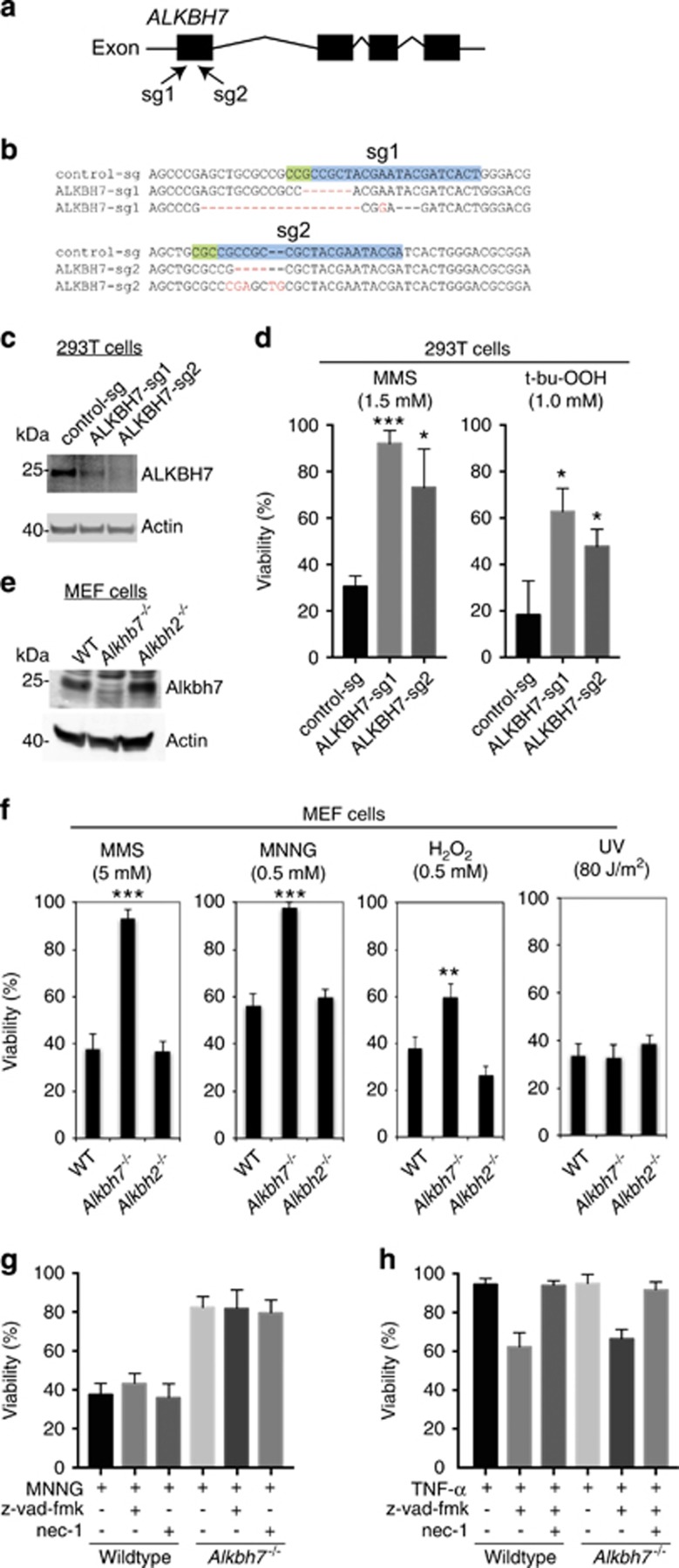
ALKBH7-deficient human or mouse cells are resistant to alkylation-induced cell death by necrosis. (**a**) Human *ALKBH7* gene structure and location of genomic sequences targeted by sgRNA-1 (sg1) and sgRNA-2 (sg2) for CRISPR-Cas9 mutagenesis. (**b**) DNA sequence of *ALKBH7* gene alleles in stable 293T cell lines generated by CRISPR mutagenesis with control-sg or ALKBH7-sg1 and sg2 RNAs. The WT sequence targeted for mutagenesis by each sgRNA is highlighted. (**c**) Immunoblot analysis of ALKBH7 levels in the indicated 293T cell lines. (**d**) Viability of control-sg and ALKBH7-sg cell lines after treatment with the indicated concentration of MMS or *t*-bu-OOH, as measured by live/dead cell staining with propidium iodide coupled with flow cytometry. (**e**) Immunoblot of extracts prepared from WT or *Alkbh7*^*−/−*^ MEF cells probed against mouse ALKBH7. (**f**) WT or *Alkbh7*^*−/−*^ MEF cells were treated with the indicated agents followed by viability analysis by vital cell stain. (**g**) Viability of WT or *Alkbh7*^*−/−*^ MEF cells after treatment with MNNG in the absence or presence of either z-vad-fmk or nec-1. (**h**) Viability of WT or *Alkbh7*^*−/−*^ MEF cells after treatment with TNF-*α* in the absence or presence of z-vad-fmk or nec-1. For (**d**), (**f**) and (**g** and **h**); error bars represent the S.D. of three, four and two independent experiments, respectively. *P<0.05; **P<0.01; ***P<0.001

To determine if ALKBH7 is necessary for regulated necrosis in mammalian cells, we used the CRISPR-Cas9 genome-editing system to introduce knockout mutations into the *ALKBH7* gene of 293T human embryonic kidney (HEK) cells. We generated cell lines containing a lentiviral vector expressing the Cas9 endonuclease along with one of two different sequence guide (sg) RNAs targeting exon 1 of the *ALKBH7* gene (ALKBH7-sg1 and sg2, Figure 1a). In addition, we generated a cell line using a lentiviral CRISPR vector targeting the *AAVS1* locus as a control for any nonspecific effects of CRISPR-Cas9 expression. The *AAVS1* locus was chosen as a wild-type (WT) control for CRISPR gene editing as it is a well-established gene locus that can be manipulated without any known effects on cell function or growth.^34^ Genotyping of the *ALKBH7* alleles in the ALKBH7-sg1 cell line revealed the presence of a six-base pair deletion and an indel frameshift mutation that is expected to reduce ALKBH7 expression (Figure 1b, ALKBH7-sg1). The ALKBH7-sg2 cell line contains a four-base pair deletion and an indel frameshift mutation in exon 1 that are expected to abolish ALKBH7 protein expression because of frameshifting and premature translation termination (Figure 1b, ALKBH7-sg2). The expected reduction or knockout of ALKBH7 expression in the ALKBH7-sg1 and sg2 cell lines, respectively, was confirmed by immunoblotting (Figure 1c).

Previously, we have shown that genotoxic stress caused by the alkylating agent, methylmethane sulfonate (MMS) or the oxidizing agent, *tert*-butyl hydroperoxide (*t*-bu-OOH), can induce regulated necrosis in HEK 293T cells.^28^ Indeed, treatment of the parental 293T or control-sg cell line with MMS led to necrotic cell death and a decrease in cell viability as judged by video microscopy and flow cytometry analysis for cells that have lost plasma membrane integrity (Supplementary Video 1-6, Figure 1d). In contrast, the ALKBH7-sg1 and sg2 cell lines were considerably more resistant to MMS treatment and exhibited a significantly higher percentage of viable cells compared with the control-sg cell line (Figure 1d, MMS). Both ALKBH7-sg1 and sg2 cell lines also displayed increased resistance to *t*-bu-OOH-induced cell death compared with the control-sg cell line (Figure 1d, t-bu-OOH). Thus, ALKBH7 is necessary for alkylation or oxidation-induced necrosis in human cells.

To determine if ALKBH7 has a role in cellular necrosis in other mammalian cell types, we next analyzed mouse embryonic fibroblast (MEF) cells derived from WT or *Alkbh7*^*−/−*^ mouse embryos.^32^ The lack of ALKBH7 expression in the *Alkbh7*^*−/−*^ MEF cells was confirmed by immunoblotting (Figure 1e). As comparison, we generated MEFs from mice deficient in *Alkbh2*, which is an AlkB homolog that catalyzes DNA repair of methyl lesions and protects against alkylation damage.^35, 36, 37^ The MEF cells were exposed to either MMS, MNNG or hydrogen peroxide (H_2_O_2_), which are known inducers of PARP1/ARTD1-dependent necrosis in MEF cells.^10, 11, 12, 13, 14, 38, 39, 40^ Similar to the results obtained with ALKBH7-deficient human cells, we find that *Alkbh7*^*−/−*^ MEF cells display increased resistance to either of the alkylating agents, MMS or MNNG, or the oxidizing agent, H_2_O_2_, relative to WT or *Alkbh2*^*−/−*^ MEF cells (Figure 1f). In contrast, both WT and *Alkbh7*^*−/−*^ MEF cells exhibit similar sensitivity to UV (Figure 1f, rightmost panel), which initiates apoptotic cell death.^41, 42^ Moreover, the inclusion of the caspase inhibitor, z-vad-fmk, or the RIP1 kinase inhibitor, necrostatin-1 (nec-1), had no significant effect on MNNG-induced necrosis in either WT or *Alkbh7*^*−/−*^ MEF cells (Figure 1g). This result indicates that ALKBH7 has a role in necrotic cell death that is distinct from apoptosis or RIP1-dependent necroptosis. To support this finding, we tested the response of WT and *Alkbh7*^*−/−*^ MEF cells after treatment with TNF-*α* and z-vad-fmk, which has previously been shown to induce RIP1-dependent necroptosis.^43, 44, 45^ Under these conditions, WT and *Alkbh7*^*−/−*^ MEF cells displayed similar levels of RIP1 kinase-dependent necroptotic cell death induced by TNF-*α* in the presence of z-vad-fmk (Figure 1h). Altogether, our results show that ALKBH7 is required for alkylation or oxidation-induced necrosis in both human and mouse cells.

### ALKBH7 deficiency confers whole-body protection to MMS in male mice

Next, we investigated the physiological role of ALKBH7 in modulating alkylation-induced necrosis *in vivo* using WT or homozygous *Alkbh7*^*−/−*^ mice. To provide an approximate LD_50_, animals were injected with a single dose of MMS (with each dose increasing in 50% increments) and monitored for 30 days as performed previously (See Materials and methods section).^46, 47^ To normalize the dose of MMS based upon body mass, mice were weighed immediately before injection. Although male mice of either genotype were heavier relative to female mice, no difference in weight was detected between WT or *Alkbh7*^*−/−*^ mice of the same sex at the time of MMS injection (Supplementary Fig. 1). All cases of toxicity occurred within 8–24 h post-MMS injection while any individuals who survived beyond the 24-h time point exhibited no changes in morbidity within the 30-day monitoring period.

**Figure 2:**
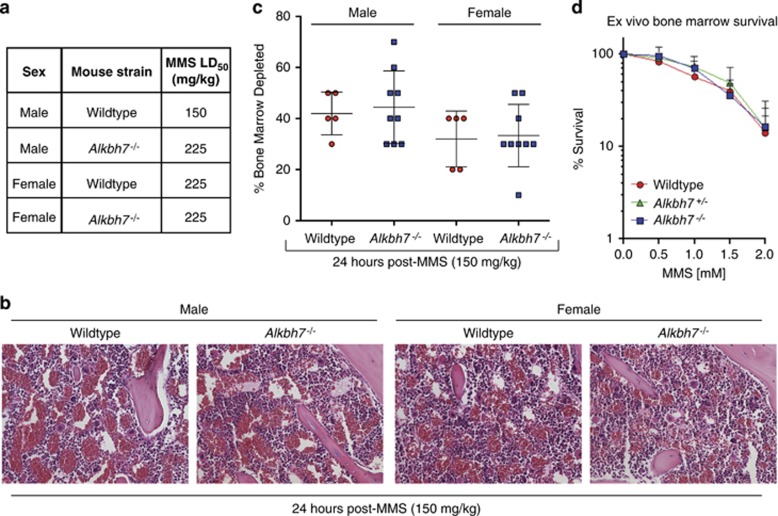
Sex-specific effects of ALKBH7 deficiency on whole-body MMS-toxicity. (**a**) Approximate LD_50_ of the indicated mouse strains in response to MMS. Four independent studies were performed. (**b**) Representative images (20X) of bone marrow samples extracted from femurs 24 h following MMS treatment (150 mg/kg). (**c**) Depletion of bone marrow is indistinguishable between WT and *Alkbh7*^*−/−*^ mice following MMS-induced damage. The percent bone marrow depletion was determined by pathological examination of bone marrow samples as shown in (**b**). (**d**) *Ex vivo* bone marrow clonogenic survival assays were performed using bone marrow isolated from WT (*n*=6), *Alkbh7*^*+/−*^ (*n*=6) and *Alkbh7*^*−/−*^(*n*=6) mice. Data represent the mean and S.D. For each of the genotypes, three male and three female mice were used in the experiments

Consistent with our previous results,^48, 49, 50^ the approximate LD_50_ for male WT mice was 150 mg/kg MMS, whereas female WT mice displayed an approximate LD_50_ of 225 mg/kg MMS (Figure 2a). Notably, we found that male *Alkbh7*^*−/−*^ mice displayed an increased tolerance to the toxic effects of MMS relative to male WT mice (Figure 2a). Although the MMS LD_50_ of male WT mice was 150 mg/kg, that for male *Alkbh7*^*−/−*^ mice was 225 mg/kg MMS. In contrast, we detected no difference in the MMS LD_50_ between female WT and female *Alkbh7*^*−/−*^ mice. In addition, we tested whether WT or *Alkbh7*^*−/−*^ mice exhibit any difference in their weight after MMS treatment but found no significant change between each genotype of the same sex (Supplementary Table 1,Supplementary Figure 2). Our results indicate that ALKBH7 deficiency in male mice confers increased resistance to the toxic effects of MMS and uncover a sex-dependent role for ALKBH7 in the response to alkylating agents.

Chemotherapeutic alkylating agents are known to cause bone marrow toxicity and myelosuppression through cell death of hematopoietic precursors and differentiated mature bone marrow cells.^51, 52, 53^ Moreover, we have previously shown that mouse bone marrow is highly sensitive to alkylation-induced cytotoxicity.^48, 49, 54, 55^ Indeed, we observed that bone marrow cellularity in WT or *Alkbh7*^*−/−*^ mice was decreased as early as 2 h after MMS treatment with continuing depletion of red blood cells, megakaryocytes and myeloid precursors throughout the MMS exposure period (data not shown). This ablation of the bone marrow remained indistinguishable between WT and *Alkbh7*^*−/−*^mice at 24 h with no detectable difference in the percentage of bone marrow depletion (Figures 2b and c, ANOVA, F(1, 24)=0.1537, *P=*0.6985). Moreover, no significant difference in bone marrow MMS sensitivity was detected between WT and *Alkbh7*^*−/−*^ mice when separated by sex (male WT *versus* male *Alkbh7*^*−/−*^, *P=*1.0; female WT *versus* female *Alkbh7*^*−/−*^, *P=*1.0). In agreement with these findings, we found that heterozygous *Alkbh7*^*+/−*^ and homozygous *Alkbh7*^*−/−*^ bone marrow cells displayed similar sensitivity to MMS-induced damage relative to WT bone marrow cells using *ex vivo* clonogenic survival assays (Figure 2d). Our studies suggest that the differences in whole-body response of *Alkbh7*^*−/−*^mice are due to changes in alkylation-sensitive tissues outside of bone marrow.

### ALKBH7 mediates MMS-induced toxicity in retina photoreceptors

Based upon our finding that male *Alkbh7*^*−/−*^mice display increased resistance to MMS-induced toxicity without changes in bone marrow toxicity, we focused on additional tissues known to be exquisitely sensitive to alkylating agent exposure, namely the retina and cerebellum.^49, 56^ We previously established that following damage induced by MMS, the cytotoxicity within the outer nuclear layer (ONL) of the retina and cerebellar granular neurons (CGNs) is caused by intermediates of the base excision repair (BER) pathway initiated by AAG and mediated through PARP1/ARTD1.^49, 56^ Retinal degeneration caused by MMS treatment is specific to the photoreceptors (rods and cones) in the ONL, whereas the adjacent inner nuclear layer and the ganglion cell layer are refractory to MMS-induced cell death.

**Figure 3:**
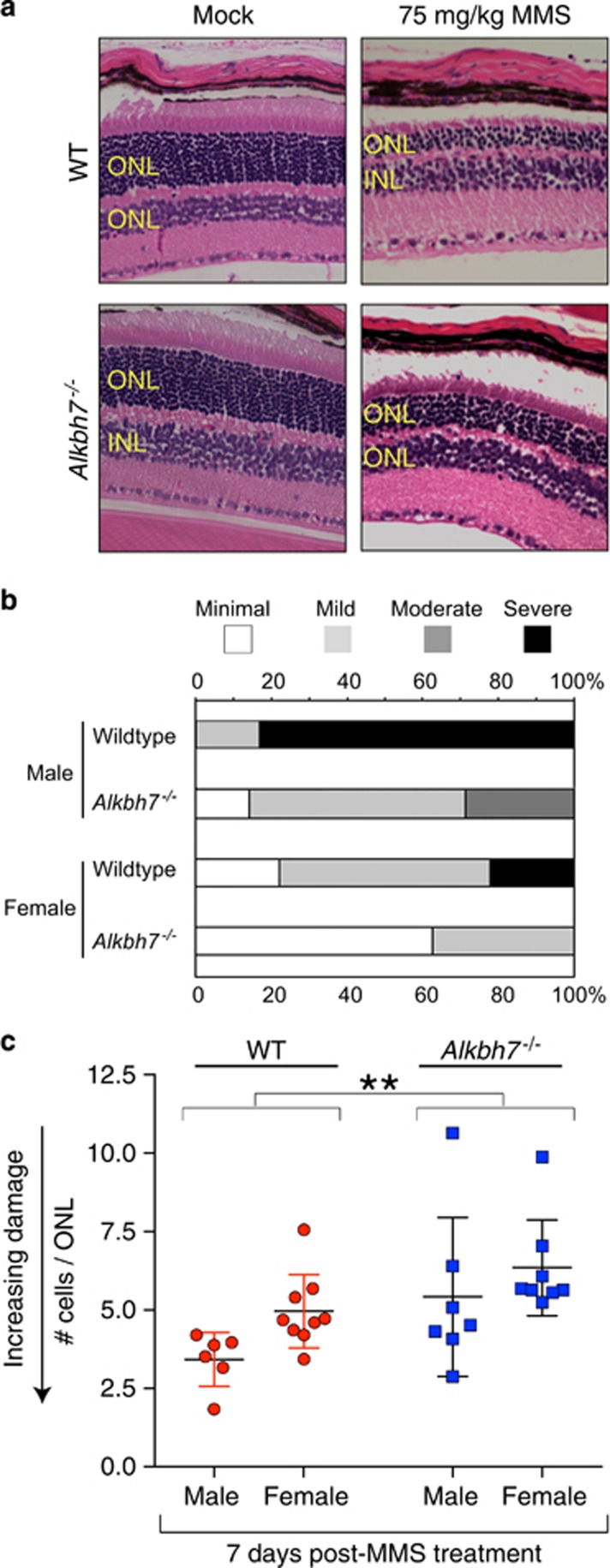
*Alkbh7* deficiency confers partial resistance against MMS-induced toxicity in retina photoreceptors. (**a**) H&E stained representative sections of WT or *Alkbh7*^*−/−*^ retina treated with either vehicle control or 75 mg/kg MMS and harvested at day 7. INL, inner nuclear layer. (**b**) Relative damage of the ONL assessed by histopathologic examination. Samples were categorized by increasing intensity of retinal degeneration ranging from minimal to severe and analyzed by chi-squared test. (**c**) Alkylation-induced retinal degeneration as determined by the thickness of the ONL. The number of cells in the ONL were counted for 25 representative areas of four sections of H&E-stained retina of the indicated mouse strains 7 days post-injection with 75 mg/kg MMS. Plotted is the average number of cells per ONL for each individual mouse, the mean (WT=4.3, *Alkbh7*^*−/−*^=5.9, and *Aag*^*−/−*^=11.2) and S.D. for each genotype. *P<0.05; **P<0.01; ***P<0.001

To determine if ALKBH7 modulates the alkylation-induced cytotoxicity in retinal cells, a sublethal dose of 75 mg/kg MMS was administered to 8–12 weeks old WT or *Alkbh7*^*−/−*^ mice through intraperitoneal injection. No difference in the ONL was observed between WT or *Alkbh7*^*−/−*^ mice after injection with PBS saline solution (Supplementary Table 2). In contrast, we observed retinal degeneration in WT mice as exemplified by the severe reduction of the ONL in comparison with PBS-injected controls (Figure 3a). Based upon a blind, nonparametric pathological assessment, we found that *Alkbh7*^*−/−*^ mice exhibited decreased levels of damage in the ONL following alkylation exposure compared with WT mice (Figure 3b, *χ*^2^(3, *N*=30)=11.077, *P=*0.011). Interestingly, male WT mice exhibited the most severe retinal degeneration induced by MMS that was significantly reduced in male *Alkbh7*^*−/−*^ mice. Female WT mice displayed less severe retinal damage induced by MMS compared to male WT mice but the severity in retinal damage could also be reduced by *Alkbh7* deficiency (Figure 3b). Thus, *Alkbh7* deficiency confers partial protection against the MMS-induced toxicity of the ONL relative to WT mice.

As an independent measure of tissue sensitivity, we quantified the number of cells across the ONL to determine tissue degeneration in response to MMS. Although both WT and *Alkbh7*^*−/−*^ mice displayed MMS-induced loss of cellular layers in the ONL, the extent of damage was reduced in *Alkbh7*^*−/−*^ mice compared with WT mice (Figure 3c). Although we detected a significant effect for genotype (WT *versus Alkbh7*^*−/−*^, ANOVA, F(1, 26)=4.142, *P<*0.05) and sex (male *versus* female, *P<*0.01), no significant interaction was identified between the two independent variables (*P=*0.621). Thus, both sex and genotype independently contribute toward protecting against MMS-induced damage such that female mice are more resistant than male mice and *Alkbh7*^*−/−*^ mice are more resistant than WT mice to MMS-induced damage.

### *Alkbh7*^*−/−*^ mice reveal a sexually dimorphic response to alkylation-induced cerebellar degeneration

**Figure 4:**
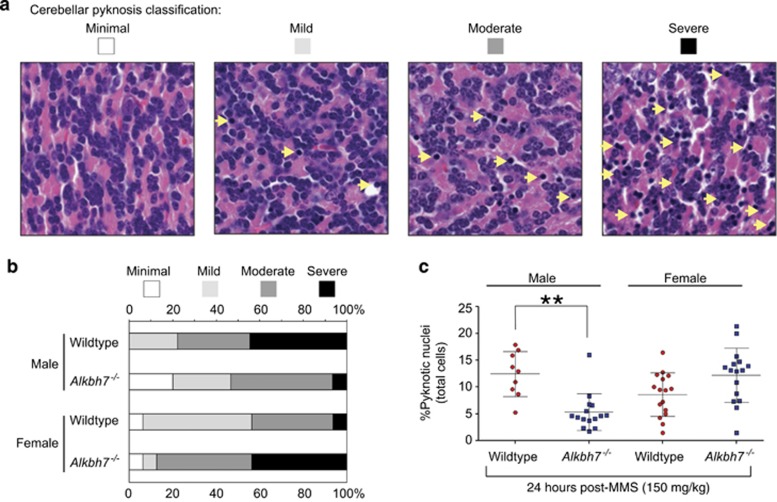
*Alkbh7*^*−/−*^ mice display a sexually dimorphic response to alkylation-induced cerebellar degeneration. (**a**) H&E-stained cerebellar granule tissue showing the spectrum of responses in mice 24 h after mock treatment (control) or injection with 150 mg/kg MMS. The relative level of damage was scored by pathological classification of CGN cell death based upon the occurrence of pyknotic nuclei (yellow arrows). (**b**) Bar graph representing the relative percentage of cerebellar pyknosis of each mouse strain after alkylation exposure as scored in (**a**). (**c**) Quantification of cellular death in the cerebellar granular layer of the indicated male or female mouse strains 24 h post-treatment with 150 mg/kg MMS. The percent pyknotic nuclei represents the average of the number of pyknotic nuclei per total number of cells across 15 fields of H&E-stained brain sections of an individual mouse strain. The mean (males: WT=12.4, *Alkbh7*^*−/−*^=5.3; females: WT=8.6, *Alkbh7*^*−/−*^=12.2) and S.D. is represented for each genotype. *P<0.05; **P<0.01; ***P<0.001

Our previous studies have shown that cerebellar granule neurons (CGNs) are a major target for necrotic cell death in response to alkylation-induced damage.^49, 56^ To determine whether ALKBH7 has a role in modulating the sensitivity of CGNs to alkylation-induced damage, we examined the cerebellum at 24 h following treatment with 150 mg/kg MMS, an amount known to rapidly cause cerebellar degeneration in WT mice.^49^ Pathological classification of cell death based on the manifestation of pyknotic nuclei revealed a spectrum of MMS-induced damage that ranged from minimal to severe (Figure 4a). Intriguingly, the severity of MMS-induced pyknotic damage in the CGNs was greatly reduced in male *Alkbh7*^*−/−*^ mice relative to male WT mice (Figure 4b). In contrast, female *Alkbh7*^*−/−*^ mice presented the highest level of cerebellar tissue damage of any mouse genotype or sex, suggesting that ALKBH7 has a sexually dimorphic role in alkylation-induced cerebellar cell death. Importantly, no major change in the number of pyknotic nuclei was detected in mice of either genotype or sex injected with PBS (Supplementary Table 3).

To further investigate the sex-specific differences in alkylation sensitivity linked to ALKBH7, we performed an independent measure of cerebellar degeneration by quantifying the percentage of pyknotic nuclei across multiple folia from sagittal sections of the cerebellum (Figure 4c). Notably, the data revealed an interaction between sex and genotype such that male *Alkbh7*^*−/−*^ male mice are rendered less sensitive to alkylation-induced cerebellar degeneration in comparison with male WT, female WT or female *Alkbh7*^*−/−*^ mice (ANOVA, F(1, 52)=21.01, *P=*2.90 × 10^-5^). In particular, we found that male *Alkbh7*^*−/−*^ mice display ~2-fold less CGN pyknosis compared with male WT mice, consistent with the pathological assessment that male *Alkbh7*^*−/−*^ mice exhibit reduced levels of alkylation-induced cerebellar degeneration (Figures 4c, *P*=0.0012). In contrast, female *Alkbh7*^*−/−*^ mice display a higher percentage of pyknotic CGNs relative to female WT mice with no significant change in resistance to alkylation-induced damage (Figure 4c, *P*=0.09). Thus, ALKBH7 deficiency protects male mice against MMS-induced death within CGNs, whereas *Alkbh7*^*−/−*^ female mice are not significantly different from either WT female or WT male mice. Altogether, our studies demonstrate a critical role for ALKBH7 in modulating a tissue-specific and sexually dimorphic cell death response that influences whole-body toxicity following exposure to an alkylating agent.

### WT or *Alkbh7*^*−/−*^ cerebellar neurons display differential MMS responses *in vitro versus in vivo*

**Figure 5:**
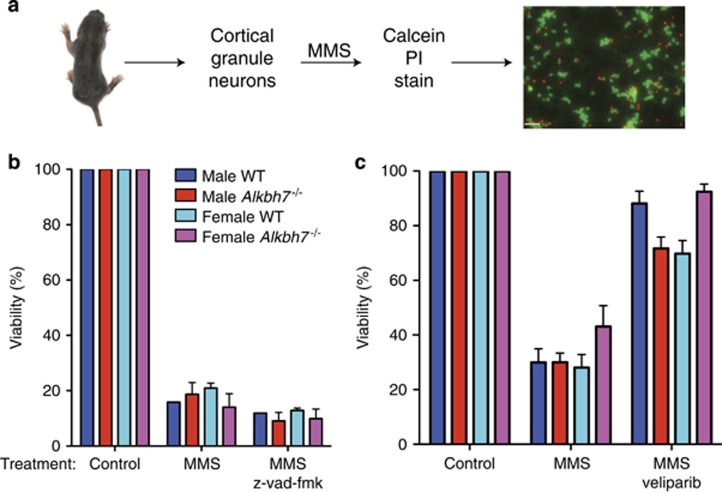
Cellular sensitivity of isolated cerebellar neurons from WT or *Alkbh7*^*−/−*^ mice in response to MMS. (**a**) Procedure for isolating cortical granule neurons (CGNs) from 6- to 8-day-old mouse pups followed by MMS treatment and viability analysis by microscopy. (**b**) Viability of the indicated CGNs after treatment with vehicle MMS or MMS with the caspase inhibitor, z-vad-fmk. (**c**) Viability of the indicated CGNs after treatment with vehicle MMS or MMS with the PARP1/ARTD1 inhibitor, veliparib. Data presented are from biological replicates, where each data point is the average of 2–4 wells with each well imaged 6–7 times

The male-specific reduction in MMS sensitivity conferred by Alkbh7 deficiency suggests sex-specific factors could be modulating necrotic cell death in concert with ALKBH7. To explore this result, we examined the MMS sensitivity of male or female CGNs isolated from 6- to 8-day-old WT or *Alkbh7*^*−/−*^ mouse pups (Figure 5a). Exposure of isolated CGNs of either sex from WT or *Alkbh7*^*−/−*^ mice led to a dose-dependent decrease in viability that was not suppressed with the caspase inhibitor, z-vad-fmk (Figure 5b). Consistent with a role for PARP1/ARDT1 in cerebellar necrosis, the survival of isolated CGNs could be rescued using the PARP1-inhibitor, veliparib (Figure 5c). Intriguingly, however, no significant difference in MMS sensitivity could be detected between WT or *Alkbh7*^*−/−*^ CGNs of either sex when analyzed *in vitro* (Figures 5b and c). These results suggest that the male-specific reduction in MMS sensitivity conferred by Alkbh7 deficiency could be related to cell non-autonomous factors that are lacking *in vitro* or absent in immature neurons.

## Discussion

Here, we show that ALKBH7 is a key factor that modulates necrotic cell death *in vivo* to influence animal toxicity after alkylating agent exposure. Intriguingly, the cell death function of ALKBH7 is specific to certain tissues such as the retina and cerebellum but has no effect on bone marrow. We also examined heart, liver and kidney tissue post-MMS treatment but detected no significant difference in tissue damage or amount of cell death between WT or *Alkbh7*^*−/−*^ mice of either sex (data not shown). We hypothesize that the tissue-specific effect of ALKBH7 on MMS toxicity is caused by differential contributions of multiple cell death pathways in each tissue type. For example, previous studies have shown that bone marrow and splenocyte cells predominantly die through a caspase-dependent apoptotic mechanism with only a minor contribution of PARP1/ARTD1-dependent necrosis.^57, 58^ Thus, the use of apoptotic rather than necrotic cell death pathways in bone marrow cells could explain the lack of any observable impact on MMS sensitivity in ALKBH7-deficient bone marrow cells.

The partial rescue of photoreceptor death in *Alkbh7*^*−/−*^ mice provides another tissue type where more than one cell death pathway could be operating to modulate tissue degeneration after alkylation exposure. Consistent with this scenario, along with others, we have shown that multiple cell death pathways contribute to retinal degeneration in response to alkylation damage, inflammation and retinal detachment injury.^49, 56, 59, 60, 61^ The necrotic cell death mechanisms that have been shown to modulate retinal cell death include PARP1/ARTD1 hyperactivation-dependent necrosis^62, 63^ and RIP-kinase-dependent necroptosis.^59, 60, 64, 65^ As the RIP-kinase-dependent pathway has been shown to be distinct from or intertwined with the PARP1/ARTD1 cell death pathway depending on the cell type and specific stress,^24, 25, 26, 66^ this could also explain why ALKBH7 deficiency confers partial rather than complete protection from alkylation-induced retinal cell death.

In addition to cell type-specific effects, we have uncovered sex-specific differences in how ALKBH7 modulates tissue responses to an alkylating agent. Although ALKBH7 contributes equally toward protecting against alkylation-induced retinal degeneration in both male and female mice, we have identified a sexually dimorphic response to alkylation-induced damage in the cerebellum. In particular, male mice deficient in ALKBH7 display increased protection against MMS-induced necrotic damage to cerebellar tissue compared with WT male mice, whereas ALKBH7 deficiency in female mice provides no protection and even sensitizes to damage. Similar to the scenarios proposed above, we hypothesize that certain tissues in female and male mice display sex-specific bias for distinct cell death pathways. Indeed, neurons that have two X-chromosomes *versus* one X and Y-chromosome display different sensitivities to cytotoxic agents with female neurons being more sensitive than male to apoptosis-inducing agents, whereas male neurons were more sensitive than female to nitrosation-induced necrotic cell death.^67^ In addition, PARP1/ARTD1 deficiency was shown to protect male but not female animals following hypoxia-ischemia stress of the brain and kidney.^68, 69, 70^ Most strikingly, protection against inflammation-induced necrosis by the absence of PARP1/ARTD1 is specific to male but not female mice.^71^ Future studies using steroids to alter sex will clarify the specific differences that contribute to the distinct responses with or without ALKBH7.

Finally, we have also found that resistance to whole-animal toxicity conferred by ALKBH7 deficiency is dependent on the sex of the mouse. In particular, male *Alkbh7*^*−/−*^ mice display increased MMS resistance relative to WT male counterparts whereas female *Alkbh7*^*−/−*^ mice display the same alkylation sensitivity as female WT mice. One potential cause for this effect is that female WT mice have achieved the maximum tolerable resistance to MMS-induced toxicity such that ALKBH7 deficiency does not confer additional protection at the whole-body level. We have previously detected such an effect with the BER enzyme, AAG, whose deficiency provides protection against MMS-induced cell death in certain tissues but does not alter the approximate LD_50_ compared with WT mice.^49^ Another potential explanation for the sex-specific protection is that a greater number of essential tissues in male mice use ALKBH7-dependent necrosis as the primary form of cell death compared with female mice. Consistent with this scenario, certain male tissues and cells use PARP1/ARTD1-dependent necrotic cell death, whereas female tissues can activate a spectrum of cell death pathways, including apoptotic mechanisms, which are independent of ALKBH7. Moreover, the sex of the mice could also affect metabolic pathways such as glycolysis or fatty acid oxidation that influences the lethal toxicity of MMS in an ALKBH7-dependent manner. Indeed, previous studies have revealed the critical role of metabolism in modulating PARP1/ARTD1-dependent necrotic cell death.^12, 38, 72^ In conclusion, our results highlight an ever-increasing role for sex in determining the alkylation response with ALKBH7 as a novel modulator of this sex-specific response. Furthermore, the sexually dimorphic response to alkylation-induced damage suggests the importance for sexual stratification in chemotherapeutic approaches that utilize alkylating agents.

## Materials and methods

### Cell culture and viability assays

293T HEK, MEF and derivative cell lines were cultured in Dulbecco's minimal essential medium (Gibco Life Technologies, Carlsbad, CA, USA) containing 10% fetal bovine serum (FBS) and 2 mM l-glutamine. MEF cells were established from day 14.5 embryos and spontaneously immortalized by serial passaging.

For viability assays, 293T HEK or MEF cells were exposed to MMS, MNNG, H_2_O_2_ or *t*-buOOH (all chemicals from Sigma, St. Louis, MO, USA) in serum-free medium for 1 h followed by replacement with medium containing serum. UV treatment was performed using a UV cross-linker (Stratagene, San Diego, CA, USA) set at a 254-nm wavelength. HEK 293T and MEF cells were analyzed 24-h post-treatment using either a Muse flow cytometer with the Muse Count and Viability Assay (EMD Millipore, Billerica, MA, USA) or a Coulter counter coupled with Trypan blue staining. For time-lapse video microscopy, cells were plated onto a 96-well plate for 24 h before treatment with 1.0 mM MMS and video capture at 20-min intervals on a Cellomics Array Scan (ThermoFisher Scientific, Waltham, MA, USA).

### CRISPR gene editing

CRISPR-Cas9 constructs were generated by cloning double-stranded oligonucleotide inserts into LentiCRISPR v2.0 (Addgene, Cambridge, MA, USA) using computationally selected genomic targets (http://crispr.mit.edu). Oligonucleotide inserts:

sg1-F: 5′-CACCGAGTGATCGTATTCGTAGCGG-3′

sg1-R: 5′-AAACCCGCTACGAATACGATCACTC-3′

sg2-F: 5′-CACCGGATCGTATTCGTAGCGGCGG-3′

sg2-R: 5′-AAACCCGCCGCTACGAATACGATCC-3′.

Lentiviral Cas9-sgRNA constructs were co-transfected with packaging plasmids (psPAX2 and pMD2.G, Addgene) into 293T cells for lentivirus production. The 293T cell line was subsequently infected with lentivirus in the presence of hexadimethrine bromide (polybrene), followed by stable clone selection using puromycin. Genotyping of the stable cell lines was performed by sequencing of cloned PCR products based upon the following primers:

A7-ex1-gPCR-F(+4)-*Bam*HI: 5′-CGCGGATCCAGGTACCGCCAATGGCTTTTGGCG-3′

A7-ex1-gPCR-R(+4)-*Hind*III: 5′-GAACTAAGCTTCGCTAAACGGTACTGTAACCGCCC-3′.

### Protein analysis

For protein extracts, human or mouse cells were harvested by trypsinzation, washed once with PBS and resuspended in Radioisotope immunoprecipitation assay buffer (50 mM TrisHCl, pH 7.5, 1% NP-40, 0.5% sodium deoxycholate, 0.1% SDS, 150 mM NaCl, 2 mM EDTA). Following a 5-min incubation on ice, cell lysates were centrifuged at 14 000 *g* at 4 °C for 15 min and the supernatant was collected for analysis. Cellular extracts were fractionated on NuPAGE Bis-Tris polyacrylamide gels (ThermoFisher Scientific) followed by transfer to Immobilon FL PVDF membrane (EMD Millipore) for immunoblotting.

Antibodies were against the following proteins: human ALKBH7 (15470-1-AP, Proteintech, Rosemont, IL, USA), mouse ALKBH7 (described in Solberg *et al.*^32^), actin in human cell extract (L00003, EMD Millipore) and actin in mouse cell extract (A5441, Sigma). Primary antibodies were detected using IRDye 800CW goat anti-mouse IgG (925-32210, ThermoFisher), IRDye 680RD goat anti-mouse IgG (926-68070, LI-COR Biosciences). Immunoblots were scanned using direct infrared fluorescence via the Odyssey System (LI-COR Biosciences, Lincoln, NE, USA).

### Animal care and treatment

*Alkbh7*^*−/−*^ and *Aag*^*−/−*^ mice were described previously.^32, 73^ All animal procedures were approved by the MIT Committee on Animal Care. Mice were housed in an AAALAC-accredited facility, fed standard chow, *ad libitum* and killed by CO_2_ asphyxiation. Tissue collection and genotyping were performed as previously described.^49, 50, 54, 56^ PCR genotyping analysis was performed using the following conditions: 95° for 5 min, 40 cycle of (95° for 1 min, 61° for 1 min, 72° for 1 min 15 s) and 72° for 10 min. PCR amplification primers: ALKBH7 For: 5′-GGGAATGGGGCTGATCTTGATGC-3′ ALKBH7 Rev WT: 5′-CATCAGACCAGCAGGATTTC-3′ and ALKBH7 Rev: 5′-CTTCAAGAGGCTTCTGTGGCAGATAAG-3′. PCR amplification results in a 650-bp band for WT and a 1030-bp band for *Alkbh7*^*−/−*^.

MMS treatments were performed by i.p. injection with varying doses of MMS (75 or 150 mg/kg). Approximate LD_50_ was determined by Deichmann and LeBlanc methodology.^47, 74^ Briefly, four independent studies were performed where two experiments tested male and female animals at the same time and the remaining two experiments tested either male or female animals alone. Two mice were treated in each experiment at the known approximate LD_50_ for WT male C57Bl/6 mice. Animals were injected with a single dose of MMS where each dose increased by 50% increments (66.6, 100, 150 and 225 mg/kg MMS) and monitored over a 30-day period. Four independent studies were performed and analysis of the approximate LD_50_ was assessed as we have previously described.^75^ At four doses per experiment, the lowest dose at which a mouse was moribund or dies within 30 days was determined as the approximate LD_50_.

### Tissue processing and histopathology

Tissues were processed by the Histology Core Facility at the David H Koch Institute for Integrative Cancer Research (Cambridge, MA, USA). Samples were paraffin-embedded, sectioned at 5 *μ*m and stained with hematoxylin and eosin (H&E). H&E slides were blindly analyzed by a pathologist (RTB) for severity of damage.

### *Ex vivo* bone marrow clonogenic assays

Bone marrow cells were harvested from the femurs of 8- to 12-week-old WT, *Alkbh7*^*+/−*^ and *Alkbh7*^*−/−*^ mice. Cells were treated for 1 h in serum-free media with the indicated doses of MMS, washed and resuspended in complete media with methylcellulose (Stem Cell Technologies, Vancouver, BC, Canada), plated in duplicate and the percent survival was calculated following 8–10 days of growth as described.^48^

### Isolation of primary CGNs and determination of cell death

CGNs were isolated from mouse pups at post-natal days 6–8 according to a previously published protocol.^76^ Each cerebellum was isolated and plated separately, representing distinct biological replicates. Cerebella were isolated and the meninges and choroid plexus were removed under a dissecting microscope in HBSS-glucose (6 g/l). The cerebellar tissue was dissociated with papain and DNase (Worthington Biochemical, Lakewood, NJ, USA) for 30 min at 37 °C, after which the tissue was further dissociated by trituration with a P1000 pipet. Cells were collected, resuspended in EBSS and pipetted atop albumin-ovomucoid inhibitor solution (Worthington Biochemical) and centrifuged for 6 min at 70 × *g*. Cells were resuspended in FBS containing neuronal media and run through a 70 *μ*m filter to remove large cells. Remaining cells were pre-plated on poly-d-lysine (PDL) coated 12-well plates (100 *μ*g/ml) for two consecutive 30-min incubations at 37°C to remove non-neuronal cells. Cells were counted and diluted with neuronal media (B27/Neurobasal+l-glutamine, penicillin/streptomycin, KCl (250 *μ*M)) to 1 × 10^6^ cell/ml. Cells were plated on PDL-coated dishes (500 *μ*g/ml) at 3000 cells/mm^2^. In all, ~95% of cells were neuronal as determined by MAP2 and GFAP immunocytochemistry (data not shown). Sex was determined by PCR using primers to amplify TSH-Beta as a control (5′-AACGGAGAGTGGGTCATCAC-3′ and 5′-CATTGGGTTAAGCACACAGG-3′) and SRY to determine sex (5′-AGAGATCAGCAAGCAGCTCC-3′ and 5′-TCTTGCCTGTATGTGATGGC-3′).

Neurons were treated with the indicated amount of MMS (Sigma) 6–8 days post-plating in neuronal media without B27 for 1 h at 37°C. Cells were pre-treated with Veliparib (5 *μ*M) for 1 h before MMS treatment and Veliparib was included in the media during and after treatment. Pan-caspase inhibitor z-Vad-fmk (Sigma) was added to cells (100 *μ*M) 3 h before treatment with MMS. Cell survival was assessed 24 h later by staining with Calcein-AM and propidium iodide (ThermoFisher Scientific) and imaged with an Array Scan XTI High Content Platform (ThermoFisher). Data presented are from biological replicates, where each data point is the average of 2–4 wells with each well imaged 6–7 times.

### Statistics

Statistical analyses were performed using IBM SPSS Statistics (IBM, Armonk, NY, USA) and GraphPad Prism 7 (GraphPad Software, Inc., La Jolla, CA, USA). Chi-square test was used to compare samples based upon categorical variables (Figures 3b and 4b). For multiple statistical comparisons based upon genotype and sex, ANOVA was performed with follow-up Tukey's tests.
