# Supplementary material for: ALKBH7 drives a tissue and sex-specific necrotic cell death response following alkylation-induced damage
Source: Cell Death Dis. 2017 Jul 20;8(7):e2947–. doi: 10.1038/cddis.2017.343 (PMC5550884; doi:10.1038/cddis.2017.343)
Supplement: Supplementary Information [file cddis2017343x1.pdf]

Supplementary Information

Supplementary Video 1. Time-lapse video microscopy of live 293T control cells after mock treatment with serum-free media for 1 hour.

Supplementary Video 2. Time-lapse video microscopy of live 293T control cells after treatment with 1.2 mM MMS in serum-free media for 1 hour.

Supplementary Video 3. Time-lapse video microscopy of live 293T control-sh cells after treatment with 1.2 mM MMS in serum-free media for 1 hour.

Supplementary Video 4. Time-lapse video microscopy of live 293T ALKBH7-sh1 cells after treatment with 1.2 mM MMS in serum-free media for 1 hour.

Supplementary Video 5. Time-lapse video microscopy of live 293T control-sh cells after treatment with 1.2 mM MMS in serum-free media for 1 hour.

Supplementary Video 6. Time-lapse video microscopy of live 293T ALKBH7-sh2 cells after treatment with 1.2 mM MMS in serum-free media for 1 hour.

Supplementary Figure 1. Weight comparison of WT and *Alkbh7*$^{-/-}$ mice

Supplementary Figure 2. Weight comparison of WT and *Alkbh7*$^{-/-}$ mice pre- and post-injection with 150 mg/kg MMS (24 hours-post).

Supplementary Table 1. Weights of WT or *Alkbh7*$^{-/-}$ mice pre- and post-injection with 150 mg/kg MMS (24 hours-post).

Supplementary Table 2. Quantification of retinal ONL cells of PBS-injected WT or *Alkbh7*$^{-/-}$ mice.

Supplementary Table 3. Quantification of pyknotic nuclei of PBS-injected WT or *Alkbh7*$^{-/-}$ mice.

Supplementary Figure 1. Weight comparison of WT and *Alkbh7*$^{-/-}$ mice. (A)(B) Mice from two independent MMS-lethal dose experiments were weighed and compared by genotype and/or sex.

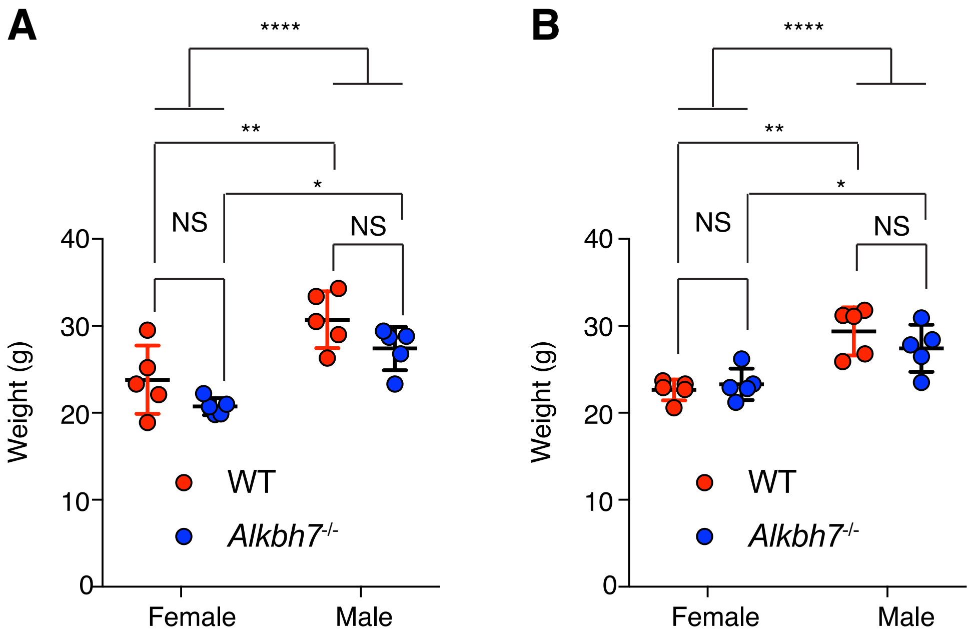

Supplementary Figure 2. Weight comparison of WT and *Alkbh7*$^{-/-}$ mice pre- and post-injection with 150 mg/kg MMS (24 hours-post).

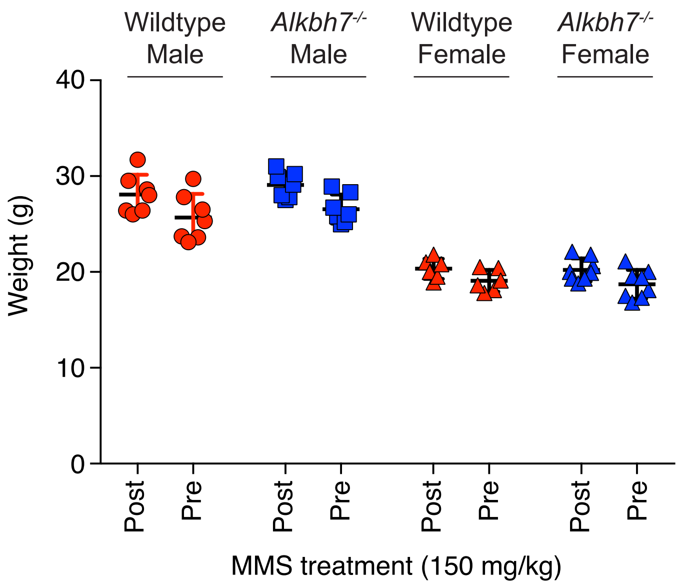

**Supplementary Table 1.**<br>**Weights of mice before and after MMS treatment (150 mg/kg), 24 hours post-injection**				
**Experiment 1**				
**Genotype**	**Sex**	**Weight before MMS (g)**	**Weight after MMS (g)**	**Change in weight after MMS**
het	male	24.5	22.78	0.930
het	male	24.7	23.52	0.952
WT	male	26.4	23.62	0.895
WT	male	26	23.73	0.913
Alkbh7$^{-/-}$	male	27.5	24.98	0.908
Alkbh7$^{-/-}$	male	27.8	25.81	0.928
Alkbh7$^{-/-}$	male	29.9	25.2	0.843
**Experiment 2**				
Alkbh7$^{-/-}$	female	22.1	20	0.905
Alkbh7$^{-/-}$	female	20.6	17.5	0.850
WT	female	21	20.5	0.976
WT	female	18.9	18.1	0.958
Alkbh7$^{-/-}$	female	21.8	21.1	0.968
Alkbh7$^{-/-}$	female	20	19.5	0.975
Alkbh7$^{-/-}$	male	28	26	0.929
Alkbh7$^{-/-}$	male	29.1	26.7	0.918
Alkbh7$^{-/-}$	male	30.2	28.3	0.937
Alkbh7$^{-/-}$	male	31	28.9	0.932
WT	male	28.6	25.3	0.885
WT	male	28	26.5	0.946
WT	male	26.4	23.1	0.875
WT	female	20	17.8	0.890
WT	female	21.8	20.4	0.936
WT	female	19.5	19.1	0.979
WT	female	20.8	18.6	0.894
Alkbh7$^{-/-}$	female	19.3	18.1	0.938
Alkbh7$^{-/-}$	female	18.8	16.8	0.894
Alkbh7$^{-/-}$	female	19.9	19.4	0.975
Alkbh7$^{-/-}$	female	19.3	17.3	0.896
WT	male	31.7	29.7	0.937
WT	male	29.5	27.8	0.942

**Supplementary Table 2. Retina ONL controls**			
**Genotype**	**Sex**	**Treatment**	**ONL thickness**
WT	Female	PBS	11.84
Alkbh7$^{+/-}$	Female	PBS	10.96
Alkbh7$^{-/-}$	Female	PBS	10.44
WT	Male	PBS	10.9
Alkbh7$^{+/-}$	Male	PBS	11.4
Alkbh7$^{-/-}$	Male	PBS	11.0

Supplementary Table 3. Pyknotic Nuclei from control (PBS-injected) cerebellum (control)							
**Genotype**	**Sex**	**Treatment**	**Pyknotic Nuclei**	**Normal nuclei**	**Total cells**	**Avg. Pyknotic Nuclei***	**%Pyknotic nuclei**
WT	Male	PBS	0	2761	2761	0	0
Alkbh7-/-	Male	PBS	13	2472	2485	1.63	0.52
Alkbh7+/-	Male	PBS	0	2560	2560	0	0
Alkbh7+/-	Male	PBS	2	2723	2725	0.25	0.07
WT	Female	PBS	0	3058	3058	0	0
Alkbh7-/-	Female	PBS	2	2646	2648	0.25	0.08
Alkbh7-/-	Female	PBS	0	2327	2327	0	0
WT	Female	PBS	5	2397	2402	0.63	0.21
Alkbh7-/-	Female	PBS	5	2382	2387	0.63	0.21
WT	Female	PBS	2	2380	2382	0.25	0.08
WT	Female	PBS	2	2180	2182	0.25	0.092

*per field, 15 fields
